# Screening for broad-spectrum antimicrobial endophytes from *Rosa roxburghii* and multi-omic analyses of biosynthetic capacity

**DOI:** 10.3389/fpls.2022.1060478

**Published:** 2022-11-16

**Authors:** Hong Zhang, Mao-Fa Yang, Qian Zhang, Bin Yan, Yu-Lan Jiang

**Affiliations:** ^1^ Department of Plant Pathology, College of Agriculture, Guizhou University, Guiyang, China; ^2^ Guizhou Academy of Testing and Analysis, Guiyang, China; ^3^ Institute of Entomology, Guizhou University, Guiyang, China; ^4^ College of Tobacco Science, Guizhou University, Guiyang, China

**Keywords:** biological activity, *Epicoccum*, genomics, non-target metabolomics, comparative genomics, *Epicoccum latusicollum*, *Setophoma terrestris*

## Abstract

Plants with certain medicinal values are a good source for isolating function-specific endophytes. *Rosa roxburghii* Tratt. has been reported to be a botanical source of antimicrobial compounds, which may represent a promising candidate for screening endophytic fungi with antimicrobial potential. In this study, 54 endophytes were isolated and molecularly identified from *R. roxburghii*. The preliminary screening using the plate confrontation method resulted in 15 different endophytic strains showing at least one strong inhibition or three or more moderate inhibition against the 12 tested strains. Further re-screening experiments based on the disc diffusion method demonstrated that *Epicoccum latusicollum* HGUP191049 and *Setophoma terrestris* HGUP190028 had excellent antagonistic activity. The minimum inhibitory concentration (MIC) test for extracellular metabolites finally indicated that HGUP191049 had lower MIC values and a broader antimicrobial spectrum, compared to HGUP190028. Genomic, non-target metabolomic, and comparative genomic studies were performed to understand the biosynthetic capacity of the screened-out endophytic fungus. Genome sequencing and annotation of HGUP191049 revealed a size of 33.24 megabase pairs (Mbp), with 24 biosynthetic gene clusters (BGCs), where the putative antimicrobial compounds, oxyjavanicin, patulin and squalestatin S1 were encoded by three different BGCs, respectively. In addition, the non-targeted metabolic results demonstrated that the strain contained approximately 120 antimicrobial secondary metabolites and was structurally diverse. Finally, comparative genomics revealed differences in pathogenicity, virulence, and carbohydrate-active enzymes in the genome of *Epicoccum* spp. Moreover, the results of the comparative analyses presumed that *Epicoccum* is a promising source of antimicrobial terpenes, while oxyjavanicin and squalestatin S1 are antimicrobial compounds shared by the genus. In conclusion, *R. roxburghii* and the endophytic HGUP191049 isolated from it are promising sources of broad-spectrum antimicrobial agents.

## 1 Introduction

Endophytic fungi are generally recognized as a group of microorganisms that do not cause substantial damage to the host and live harmlessly in healthy plant tissues throughout a certain life cycle stage (Yuan et al., 2018). The existence of fungi inside the tissues of healthy plants has been known as early as the late 19th century when endophytic fungi were first successfully isolated from darnel (*Lolium temulentum*) (Freeman, 1904; Kusari et al., 2012). However, plant endophytic fungi had not attracted much attention as a new microbial resource until 1993. When an endophytic fungus, Ceriporiopsis andreanae (basionym Taxomyces andreanae), was isolated from Taxus brevifolia for the production of taxol, which initiated a surge in studies on endophytes (Stierle et al., 1993; Cheng et al., 2022). Another excellent anticancer drug, vincristine is originally reported from *Catharanthus roseus*, endophytic *Fusarium oxysporum* isolated from this plant can also produce vinblastine and vincristine in appreciable amounts (Kumar et al., 2013). The herb *Artemisia annua* L. is well known for its antimalarial properties and is the source of the antimalarial drug artemisinin (Madsen et al., 2010). Extracts of both endophytic *Penicillium* and *Talaromyces* isolated from *A. annua* exhibited significant antimalarial activity (Alhadrami et al., 2021). Therefore, endophytic fungi can produce the same natural products as their host plants. Importantly, microbial fermentation has several advantages over the use of plants for the production of bioactive substances, such as easy-to-operate, reducing the need for plants, and obtaining stronger active drug derivatives by varying the culture conditions (Kumar et al., 2013).

Various endophytic fungi have been employed in recent years to produce bioactive compounds, such as *Aspergillus*, *Epicoccum*, *Hypoxylon*, *Induratia*, *Penicillium*, *Phoma*, *Phaeosphaeria*, *Saccharomycopsis*, *Sarocladium*, *Trichoderma*, and *Wickerhamomyces*. The biologically active secondary metabolites deriving from endophytic fungi belong to diverse structural classes. The secondary metabolites include alkaloids, anthraquinones, polyketides, sterols, terpenes, and volatile organic compounds (Zhang et al., 2021a). They possess potent antimicrobial, antiviral, insecticidal, antioxidative, antidiabetic, cytotoxic, and anticancer properties (Deshmukh et al., 2015; Zhang et al., 2019; Fernando et al., 2020; Manganyi and Ateba, 2020; Pal et al., 2020; Rahaman et al., 2020; Agrawal et al., 2022). A few endophytic fungi can produce phytohormones to promote the growth of their host plants. And synthesize bioactive compounds to increase the resistance of the plants to environmental stresses. Still, they can also promote the accumulation of secondary metabolites initially produced by the plant, including pharmaceutical ingredients (Jia et al., 2016).

Research on endophytic fungi has become more accessible with the continual advancement of sequencing and omics technologies. Genetics- and genomics-based strategies have emerged as a comprehensive approach to studying natural microbial products (Walker et al., 2020). It is possible to elucidate the basic pathways of secondary metabolites isolated from organisms using these technologies. These technologies can facilitate the computational discovery of biosynthetic pathways. Producer strains for biosynthesis are investigated, silenced biosynthetic gene clusters are activated, and synthetic pathways for novel compounds are designed to increase their yields and activity (Sagita et al., 2021). Comparative multi-genome analysis significantly improves understanding of the genetic and metabolic diversity of endophytic fungi involved in different host-plant interactions (Ye et al., 2017). Additionally, the putative functional characteristics of endophytes can be elucidated by metagenome-based analyses (Gupta et al., 2020). The rapid development of omics technologies has accelerated the development of endophytic fungal resources.

*Rosa roxburghii* Tratt., a homology of medicine and food, has received considerable attention across many research fields because of its notably high vitamin C. Various phytochemicals extracted from its fruits, roots, and leaves have shown potential antimicrobial activity. When choline chloride with lactic acid or levulinic acid (molar ratio 1:2) as deep eutectic solvents, the extracts of *R. roxburghii* leaves showed incredible antibacterial activities against the five tested pathogens (*Bacillus subtilis*, *Escherichia coli*, *Listeria monocytogenes*, *Salmonella typhimurium*, and *Staphylococcus aureus*), with the minimum inhibition concentration (MIC) values ranging from 0.012 to 0.049 mg/mL (Wang et al., 2021). Strictinin isomers, separated from the root of this plant, were excellent antimicrobial components, mainly responsible for oxidative stress and protein synthesis disorder (Ma et al., 2020). Since *R. roxburghii* is a botanical source of antimicrobial compounds, it may represent a promising target for screening endophytic fungi with antimicrobial potential. We isolated endophytic fungi from *R. roxburghii* and screened the most prospective strain by antimicrobial activity assays. Additionally, we investigated the strain’s biosynthetic capacity through genomics, non-targeted metabolomics, and comparative genomics.

## 2 Materials and methods

### 2.1 Isolation and identification

#### 2.1.1 Sample collection and endophyte isolation

Healthy *R. roxburghii* tissues (roots, stems, leaves, flowers, fruits, and seeds) were collected from April to August 2020 in Guizhou Province (27°4′50″ N, 106°29′50″ E and 25°52′52″ N, 104°33′59″ E), China. Endophytic fungi were isolated from different tissue parts using a surface sterilization method (Wang et al., 2019). The main steps of the procedure: Tissue segments were surface-sterilized with 75% ethanol for 1 min, rinsed thrice with sterile water, immersed in 1% (w/v) aqueous sodium hypochlorite (NaOCl) for 1–3 minutes (roots, 2 min; stems and seeds, 3min; and leaves, fruits, and flowers, 1 min), and washed thrice again with sterile distilled water. Six different media were used for fungal isolation, namely, potato dextrose agar (PDA), oatmeal agar (OA), malt extract agar (MEA), Czapek Dox agar (CDA), water agar (WA), and synthetic low nutrient agar (SNA). The media were supplemented with streptomycin sulphate (0.5 g/L) to avoid bacterial contamination. Meanwhile, the effectiveness of surface sterilization was examined according to the previous description (Singh et al., 2017; Rojas et al., 2020). All pure isolates were stored at -80°C with 30% glycerol.

#### 2.1.2 Molecular identification

DNA was extracted from mycelia grown on potato dextrose agar (PDA) according to the manufacturer’s instructions for a Fungal gDNA Isolation Kit (BW-GD2416, Biomiga, China). The primers used for polymerase chain reaction (PCR) amplification and sequencing included ITS5/ITS4 for ITS (White et al., 1990), LR0R/LR5 for LSU (Vilgalys and Hester, 1990), Bt2a/Bt2b for TUB (Glass and Donaldson, 1995), and fRPB2-5F/fRPB2-7cR for RPB2 (Liu et al., 1999). Successful amplification is generally obtained by annealing at 55°C for 35 cycles. The PCR products were sequenced by Sangon Biotech (Shanghai, China).

Endophytic fungi were identified based on multigene phylogenetic analyses. Consensus sequences were edited with BioEdit v. 7.0.9.0 (Hall, 1999). Multiple sequence alignment was performed using MAFFT v. 7 (Katoh et al., 2019), manually adjusted in BioEdit, and concatenated in PhyloSuite v. 1.2.2 (Zhang et al., 2020). Phylogenetic analyses were inferred from maximum likelihood (ML) and Bayesian inference (BI). ModelFinder determined the substitution models based on the Bayesian Information Criteria (BIC) and Akaike information criterion (AIC) (Kalyaanamoorthy et al., 2017). BIC was used for ML analyses, while AIC was used for BI analyses. ML tree inference was constructed using 10,000 ultrafast bootstraps (Minh et al., 2013) under the edge-linked partition model implemented in IQ-TREE (Nguyen et al., 2014). BI analyses were carried out in MrBayes 3.2.6 (Ronquist et al., 2012) under the partition models, with two independent runs of four chains that were run for five million generations using the Markov chain Monte Carlo algorithm. Finally, the resulting trees were visualized using Figtree v.1.4.3 (Rambaut, 2014).

### 2.2 Antimicrobial activity

#### 2.2.1 Tested strains

To evaluate the antimicrobial activity of endophytic fungi, the following microorganisms were used: Six tested fungi, including kiwifruit soft rot pathogens *Lasiodiplodia theobromae* and *Botryosphaeria dothidea*, pepper anthracnose fungus *Colletotrichum capsici*, rice blast fungus *Pyricularia oryzae*, rice sheath blight fungus *Rhizoctonia solani*, and root rot fungus *Fusarium oxysporum* (causing *Pseudostellaria heterophylla* and *Zanthoxylum schinifolium* diseases). Six tested bacteria, namely kiwifruit bacterial canker pathogen *Pseudomonas syringae* pv. *actinidiae*; peach bacterial shot hole pathogen *Pantoea agglomerans*; other bacteria *Bacillus subtilis* CMCC (B) 63501, *Escherichia coli* CMCC (B) 44102, *Pseudomonas aeruginosa* ATCC 27853, and *Staphylococcus aureus* ATCC 6538.

#### 2.2.2 Preliminary screening of antimicrobial activity assay

Endophytic strains with antagonistic ability were screened out by the plate confrontation method (Gao et al., 2021). The width of the zone of inhibition (*I*) between tested fungi (or bacteria) and endophytes was determined according to the previously described method (Gao et al., 2021). The definition of the inhibition intensity is based on the previously described method (Gashgari et al., 2016; Zhao et al., 2019). The intensity is divided into four levels, which are indicated by 0, 1, 2, and 3 for no inhibition, weak inhibition, moderate inhibition, and strong inhibition, respectively. For evaluating antifungal activity: 0 (*I* = 0 mm), 1 (0 mm < *I* ≤ 1 mm), 2 (1 mm < *I* ≤ 3 mm), and 3 (*I* > 3 mm); for antibacterial activity: 0 (*I* ≤ 1 mm), 1 (1 mm < *I* ≤ 2 mm), 2 (2 mm < *I* ≤ 10 mm), and 3 (*I* > 10 mm). Through phylogenetic analyses and preliminary screening, strains were selected for re-screening antimicrobial activity under the following principles: with the best inhibition effect in the same species and strong inhibition of at least one or moderate inhibition of three or more against the tested strains.

#### 2.2.3 Secondary metabolites extraction

To further investigate the antimicrobial activity of the initially screened-out strains, crude extracts of the secondary metabolites were prepared using the method described previously (Zhang et al., 2021b). The endophytic strains were fermented in Erlenmeyer flasks (250 mL) containing 100 mL potato dextrose broth (PDB) (potato: 200 g/L, glucose: 20 g/L, and natural pH) at 28 ± 1°C, 220 rpm, and for 7–10 d. High-speed centrifugation (14,000 g, 10 min) was performed to separate the culture broth and mycelium, which were extracted by ethyl acetate (EtOAc) and methanol (MeOH)-assisted sonication, respectively. Then concentrated at 50°C under reduced pressure until constant weight and dissolved in dimethyl sulfoxide (DMSO) to obtain 20 mg/mL of EtOAc crude extract (extracellular metabolites) and MeOH crude extract (intracellular metabolites).

#### 2.2.4 Re-screening of antimicrobial activity assay

The extracellular and intracellular metabolites were re-screened for antimicrobial activity using the disc diffusion method (Hu et al., 2017; Rjeibi et al., 2020). For antifungal assay: Briefly, a tested fungal plug (6 mm diam.) and a same-sized sterile filter paper disc were placed at the appropriate position of the PDA plate (90 mm diam.). The disc was impregnated with 10 μL of metabolite (20 mg/mL). DMSO was used as a negative control. All plates were incubated at 28 ± 1°C. The radial growth of the tested strains was measured after 2–7 d. Negative control plates as *R*_1_ and experimental plates containing metabolites as *R*_2_. The percentage inhibition (%) = (*R*_1_-*R*_2_)/*R*_1_ × 100% (Hajieghrari et al., 2008).

For antibacterial assay: The sterile disc (6 mm diam.) was placed at the center of the nutrient agar (NA) plate, which had been coated with tested bacteria, and then impregnated with 10 μL of extracellular or intracellular metabolite (20 mg/mL). Equal volumes of DMSO were used as a negative control. The diameters of the inhibition zone (d) were measured after culturing for 24–48 hours at 25°C ± 1°C for phytopathogenic bacteria and 35°C ± 1°C for other tested bacteria. Similarly, the MICs were determined. The assays were repeated three times.

### 2.3 Morphological observations

Morphological characteristics were observed on PDA. In this study, the final screened-out strain with antimicrobial potential was inoculated on PDA, cultured at 28°C for 5–7 days, and then placed at 4°C for preservation to promote sporulation. Macroscopic morphology was examined under a digital microscope (VHX-7000, Keyence). After sporulation, micromorphological features and dimensions of the spores were determined in 25% lactic acid under a Zeiss Axiolab 5 light microscope equipped with an Axiocam 208 camera.

### 2.4 Genome-sequencing, annotation, and analyses

Strain with the strongest antimicrobial activity from the re-screening was selected for whole-genome sequencing to deeply analyze its biosynthetic capacity. Genomic DNA was sequenced using a combination of second-generation Illumina sequencing technologies and third-generation PacBio sequencing technology at Guangzhou Genedenovo Biotechnology Co., Ltd. The endophytic strain was grown in a 1  L Erlenmeyer flask containing 500  mL of PDB at 28°C under 220  rpm for three days. The fermentation broth was centrifuged at 14,000 g for 10 min at 4°C, the supernatant was discarded, and the mycelium was collected and used for genomic DNA extraction. Genomic DNA was extracted using commercial kits, and DNA quality was assayed using Qubit (Thermo Fisher Scientific, Waltham, MA) and Nanodrop (Thermo Fisher Scientific, Waltham, MA). Qualified genomic DNA was fragmented with G-tubes (Covaris, Woburn, MA, USA) and end-repaired to prepare SMRTbell DNA template libraries with a fragment size of >10 Kb. Then, library quality was detected by Qubit^®^ 2.0 Flurometer (Life Technologies, CA, USA), and average fragment size was estimated on a Bioanalyzer 2100 (Agilent, Santa Clara, CA). Subsequently, SMRT sequencing was performed on the Pacific Biosciences Sequel sequencer (PacBio, Menlo Park, CA) following standard protocols (MagBead Standard Seq v2 loading, 1 × 180 min movie) with the P4-C2 chemistry.

Continuous long reads attained from SMRT sequencing were corrected for random errors in the long seed reads (seed length threshold 6 Kb) by aligning shorter reads from the same library using MECAT. The resulting corrected, preassembled reads were used for *de novo* assembly using MECAT with an overlap-layout-consensus (OLC) strategy (Myers et al., 2000; Xiao et al., 2017). The Open reading frame (ORF) was predicted using the GeneMark-ES (Ter-Hovhannisyan et al., 2008). Repetitive elements were identified by RepeatMasker (Chen, 2004). Noncoding RNAs, such as rRNAs prediction, were carried out using RNAmmer (Lagesen et al., 2007), and tRNAs were identified by tRNA-scan-SE (Lowe and Eddy, 1997).

Functional annotation of predicted protein-coding genes against National Center for Biotechnology Information (NCBI) non-redundant Protein (Nr) database, Gene Ontology (GO), eukaryotic orthologous groups (KOG), Kyoto Encyclopedia of Genes and Genomes (KEGG), and SwissProt databases were conducted by the BlastP method. Moreover, the assembled genome sequence was analyzed for secondary metabolite biosynthesis gene clusters (BGCs) using antiSMASH 6.1.1.

### 2.5 Non-targeted metabolomics analyses

The PDB fermentation conditions for endophytic fungi were identical to the genomic sequencing assay. The fermentation broth of the endophyte was separated by high-speed centrifugation (14,000 g, 10 min). We selected extracellular or intracellular metabolites with better inhibitory activity for non-target metabolomics analyses based on the results of the re-screening assay. The culture broth samples were thawed at 4°C, and 100 μL aliquots were mixed with 400 μL of cold methanol/acetonitrile/H_2_O (2:2:1, v/v/v). Following vortex mixing, low-temperature sonication for 30 min and resting for 10 min at -20°C. After that, the mixture was centrifuged for 20 min (14,000 g, 4°C). The supernatant was dried in a vacuum centrifuge. The samples were re-dissolved in 100 μL acetonitrile/water (1:1, v/v) for LC-MS analyses. Analyses were performed using a UHPLC (1290 Infinity LC, Agilent Technologies) coupled to a quadrupole time-of-flight (AB Sciex Triple TOF 6600). The chromatographic separation and The ESI source conditions were as previously described (Huang et al., 2019).

### 2.6 Comparative genomics analyses

Relevant genomic data released by NCBI were selected for comparative genomic profiling. Three *de novo* gene prediction programs, Augustus v.2.7, GeneMark+ES v.4.0, and SNAP v.2013-02-16, were used to predict the protein-coding regions if only genomic data were available in NCBI. The Maximum likelihood tree of genomes was performed using single-copy orthologous genes. Pathogen-host interaction (PHI), carbohydrate-active enzymes (CAZymes), and BGCs were annotated using PHI-base v. 4.13, dbCAN2 v. 11, and antiSMASH v. 6.1.1, respectively. Genes encoding BGCs were aligned using MAFFT v. 7, the substitution model was determined by ModelFinder, and ML tree inference was performed in IQ-TREE using 10,000 ultrafast bootstraps. Finally, the annotation results of each genome were compared and analyzed.

### 2.7 Data analysis

Data were analyzed by ANOVA, followed by comparisons of means using the LSD test in Data Processing System (DPS v9.50) (P < 0.05).

## 3 Results

### 3.1 Identification of endophytic fungi

**Figure 1:**
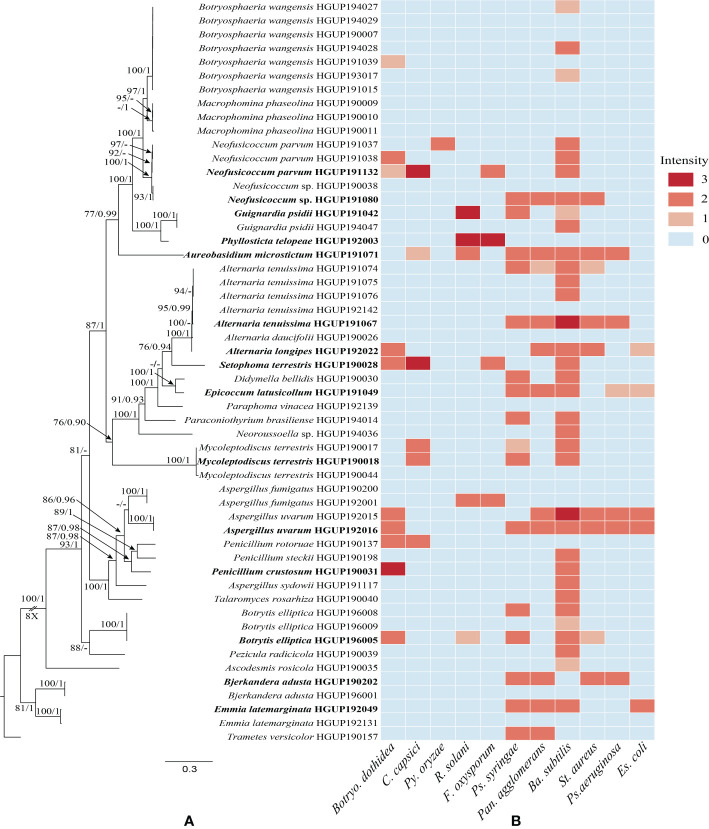
Phylogenetic relationships and preliminary screening for antimicrobial activity of endophytic fungi. **(A)** Phylogram generated from maximum likelihood (ML) analyses, based on combined ITS, LSU, TUB, and RPB2 sequence data. Bootstrap support values for ML greater than 75% and Bayesian posterior probabilities greater than 0.90 are given near nodes, respectively. Bold indicates strains that have been preliminarily screened out. **(B)** Heatmap of antimicrobial activity spectra against the tested strains.

All culturable endophytic strains were sequenced and used for multigene phylogenetic analyses. 54 strains belonging to Dothideomycetes, Eurotiomycetes, Pezizomycetes, Leotiomycetes, and Agaricomycetes were successfully isolated and identified from tissue segments of *R. roxburghii*. The largest number of endophytes was found in root tissues (20 isolates), followed by the stem (14 isolates), leaf (9 isolates), fruit (6 isolates), seed (4 isolates), and flower (one isolate). Of these isolates, 51 strains were identified at the species level, covering 28 confirmed species. The remaining genus-level isolates, including two unidentified species, may belong to new taxa. The phylogenetic relationship was constructed with combined ITS, LSU, TUB, and RPB2, as illustrated in **Figure 1A**.

### 3.2 Preliminary screening results

Preliminary screening results of the 54 isolates for antimicrobial activity *in vitro* were represented in **Figure 1B** and **Supplementary Table 1**. In this study, the antibacterial activity of endophytic fungi was superior to the antifungal activity. The antimicrobial activity may be strain-specific owing to significant differences observed among strains of the same endophytic species, such as *Alternaria tenuissima*, *Emmia latemarginata*, and *Neofusicoccum* sp. Most endophytes exhibited broad-spectrum activities, whereas another small group did not display any antimicrobial activity, e.g., *Macrophomina phaseolina* and *Paraphoma vinacea*. Concretely, endophytes showed stronger antibacterial activity against *Ba. Subtilis*, and hardly any activity were observed against *Py. oryzae*. Although generally described as pathogens, some species as endophytic fungi also demonstrated potential antimicrobial activity, e.g., *Al. tenuissima* HGUP191067. In general, 15 endophytes were selected for subsequent experiments based on molecular identification and the strength of inhibition activity (**Figure 1**).

### 3.3 Re-screening of antimicrobial activity

#### 3.3.1 Re-screening of antifungal activity

**Figure 2:**
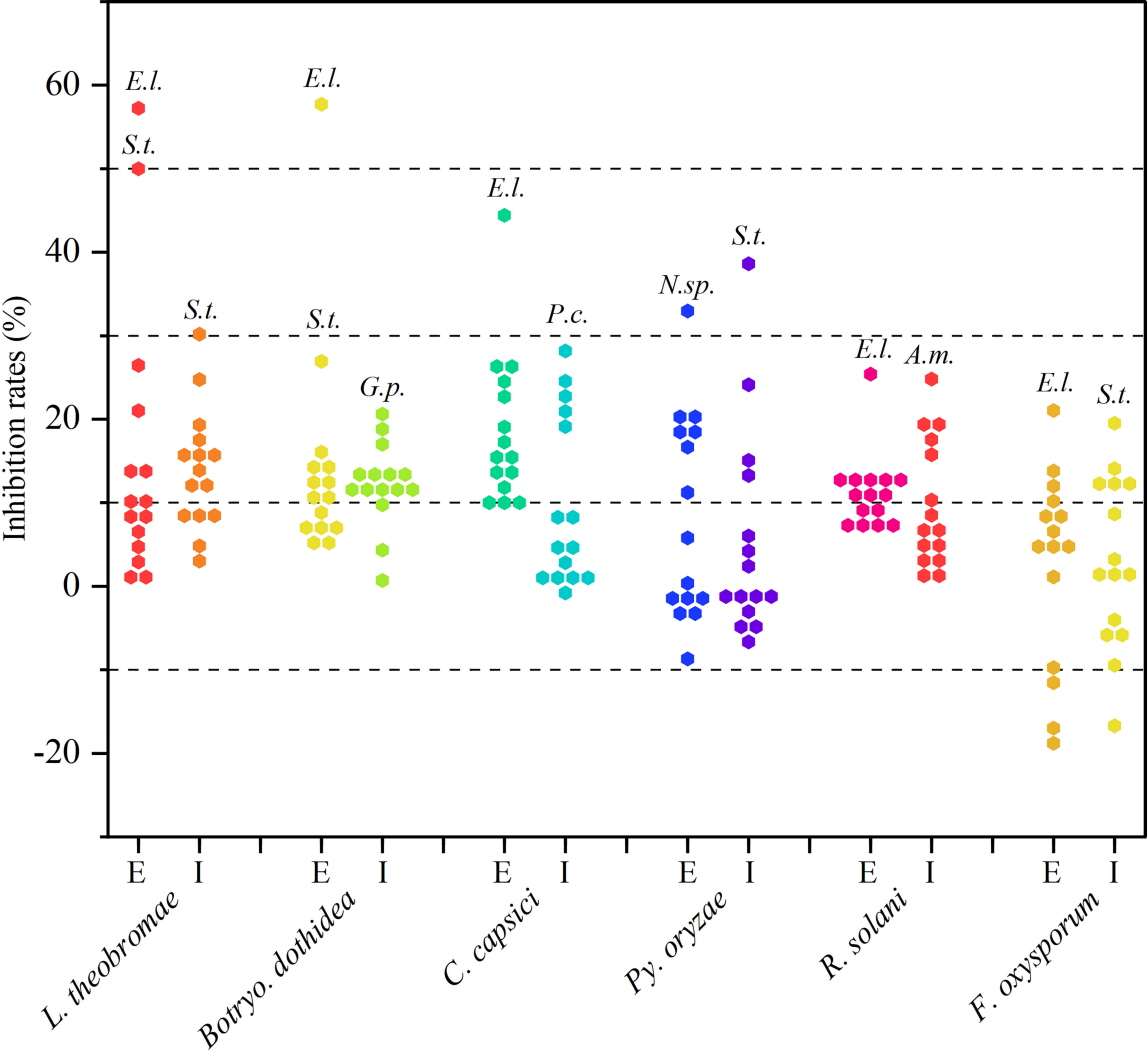
Results of re-screening for antifungal activity. E and I indicate extracellular and intracellular metabolites, respectively. *A.m.*, *E.l.*, *G.p.*, *N.sp.*, *P.c.*, and *S.t.*, represent *Aureobasidium microstictum* HGUP191071, *Epicoccum latusicollum* HGUP191049, *Guignardia psidii* HGUP191042, *Neofusicoccum* sp. HGUP191080, *Penicillium crustosum* HGUP190031, and *Setophoma terrestris* HGUP190028, respectively.

As observed from the trends of **Figure 2** and **Supplementary Table 1**. These extracellular and intracellular metabolites, which were prepared from the preliminary screened-out strains, showed broad-spectrum antifungal activity against at least one phytopathogenic fungus. However, most metabolites exhibited less than 20% inhibition against the six tested fungi. Fortunately, endophytic strains *Epicoccum latusicollum* HGUP191049, *Neofusicoccum* sp. HGUP191080, and *Setophoma terrestris* HGUP190028 displayed potential as antifungal agents since their metabolites displayed over 30% inhibition rate against at least one of the tested fungi. Of these, the extracellular metabolites of HGUP191049 and HGUP190028 were highly effective against *L*. *theobromae* with inhibition rates was 58.5 ± 3.4% and 51.4 ± 3.4%, respectively. Meanwhile, the inhibition rate of HGUP191049 also reaches 58.0 ± 2.2% against *Botryo. dothidea* and 45.3 ± 1.3% against *C. capsici*. Accordingly, *Ep. latusicollum* HGUP191049 holds good promise for developing antifungal agents.

#### 3.3.2 Re-screening of antibacterial activity

**Figure 3:**
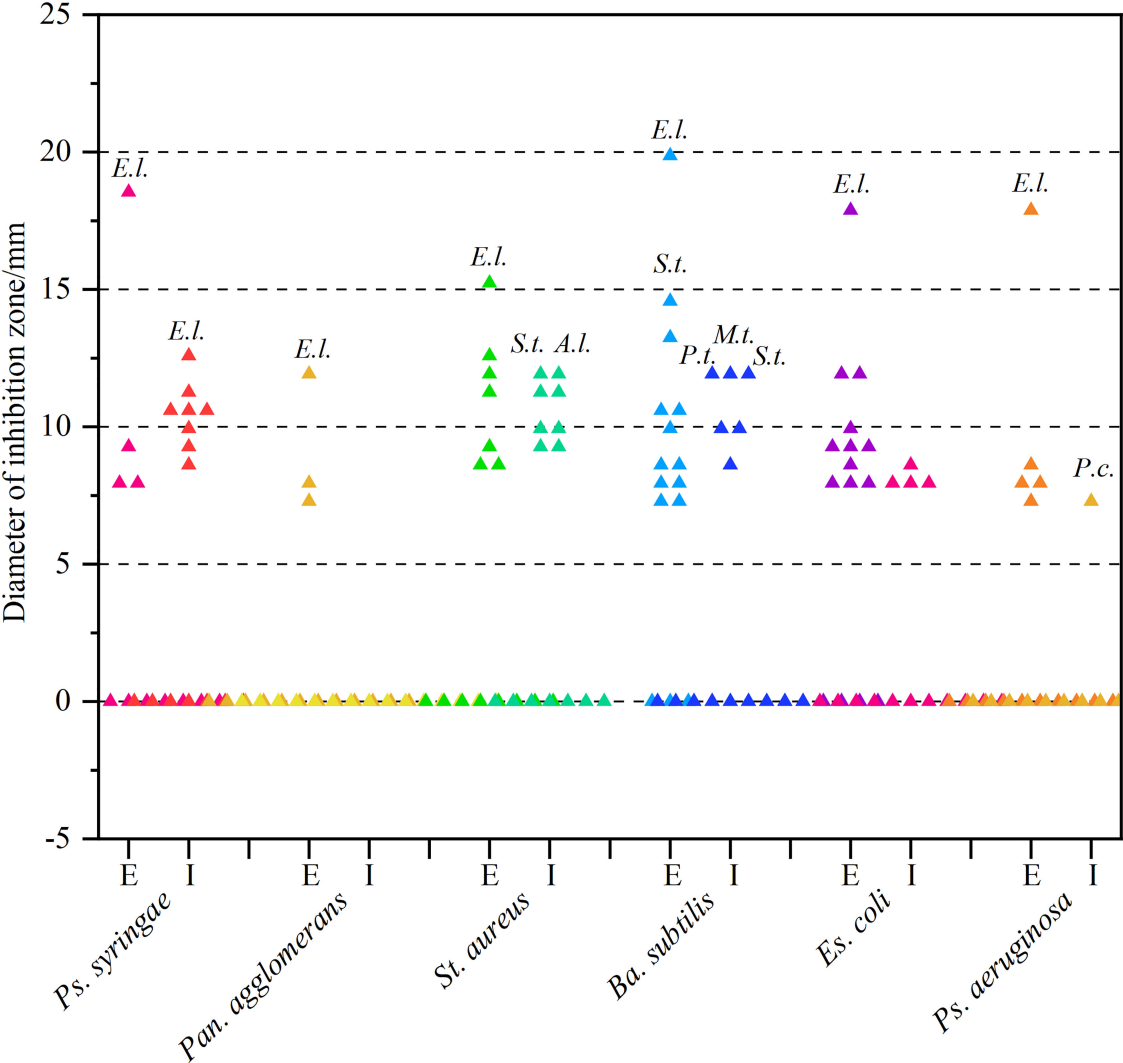
Results of re-screening for antibacterial activity. E and I indicate extracellular and intracellular metabolites, respectively. *A.l.*, *E.l.*, *M.t.*, *P.c.*, *P.t.*, and *S.t.* stand for *Alternaria longipes* HGUP192022, *Epicoccum latusicollum* HGUP191049, *Mycoleptodiscus terrestris* HGUP190018, *Penicillium crustosum* HGUP190031, *Phyllosticta telopeae* HGUP192003, and *Setophoma terrestris* HGUP190028, respectively.

As can be derived from **Figure 3** and **Supplementary Table 1**, most secondary metabolites showed sub-moderate inhibition intensity (d < 10 mm). However, the extracellular metabolite of *Ep. Latusicollum* HGUP191049 was shown to have potent antibacterial activity against both tested Gram-positive (*St*. *aureus* and *Ba*. *subtilis*) and Gram-negative (*Ps. syringae* pv. *actinidiae*, *Es. coli*, and *Ps. aeruginosa*) bacteria, since inhibition zone diameters ranging from 15.3 ± 1.5 mm to 20.3 ± 2.5 mm. So the strain HGUP191049 was considered to be well antagonistic.

#### 3.3.3 Determination of the MIC

Two endophytic isolates *Ep. latusicollum* HGUP191049 and *Se. terrestris* HGUP190028 had a better antimicrobial effect in the re-screening assay based on a broad spectrum and intensity. To evaluate the antimicrobial potential of the extracellular metabolites of the two isolates, in which MIC values were determined. As shown in **Table 1**, *Ep. latusicollum* HGUP191049 presented MIC values of 1.25 mg/mL, 2.50 mg/mL, and 1.25 mg/mL against *L. theobromae*, *Botryo. Dothidea*, and *R. solani*, respectively, whereas MIC values ranged from 0.31 mg/mL to 5.00 mg/mL against six tested bacteria. The endophytic strain HGUP191049 had more potential for antimicrobial properties than the strain HGUP190028.

**Table 1:** The minimum inhibitory concentration (MIC) of extracellular metabolites of two endophytic isolates against 12 tested strains.

Species	Strain no.	MIC concentration (mg/mL)					
		*L*. *theobromae*	*Botryo*. *dothidea*	*C*. *capsici*	*Py*. *oryzae*	*R*. *solani*	*F. oxysporum*
*Setophoma terrestris*	HGUP190028	5.00	5.00	10.00	20.00	–	10.00
*Epicoccum latusicollum*	HGUP191049	1.25	2.50	10.00	–	1.25	10.00
**Species**	**Strain no.**	***Ps. syringae***	***Pan. agglomerans***	***St. aureus***	***Ba*. *subtilis***	***Es*. *coli***	***Ps. aeruginosa***
*Setophoma terrestris*	HGUP190028	–	–	2.50	0.31	10.00	–
*Epicoccum latusicollum*	HGUP191049	0.31	1.25	5.00	0.62	2.50	2.50

### 3.4 Taxonomy of *Epicoccum latusicollum*

**Figure 4:**
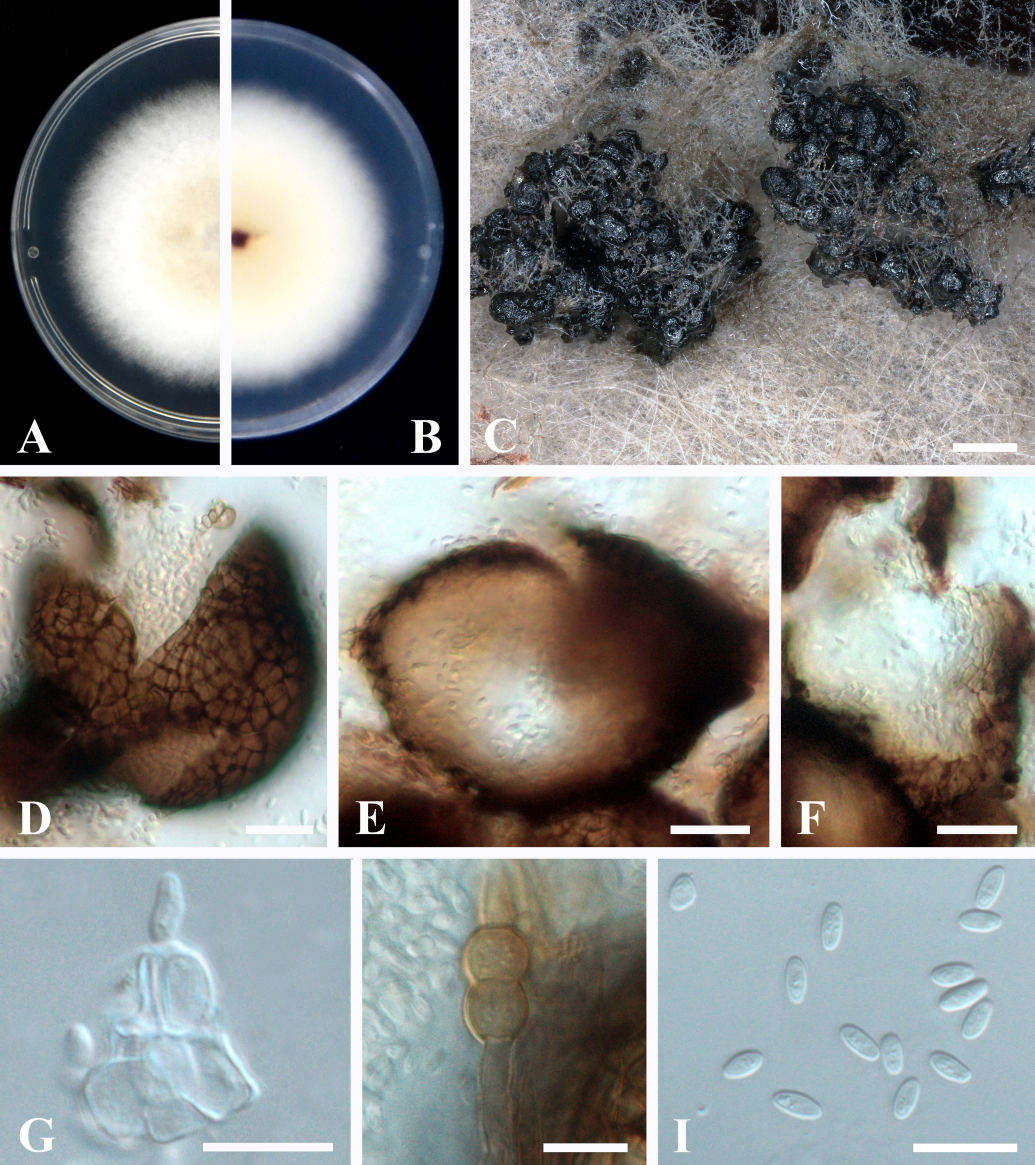
*Epicoccum latusicollum* (HGUP191049). **(A, B)**. Colony on PDA (front and reverse). **(C)** Pycnidia forming on PDA. **(D)** Pycnidia. **(E)** Section of pycnidium. **(F)** Section of pycnidial wall. **(G)** Conidiogenous cells. **(H)** Chlamydospores. **(I)** Conidia. Scale bars: **C** = 200 μm; **D**–**F** = 20 μm; **G**–**I** = 10 μm.

Sexual morph not observed. Asexual morph (**Figure 4**): Conidiomata pycnidial, aggregated, superficial, black, globose to subglobose or pyriform, glabrous, up to 140 μm diam., without distinct ostioles. Pycnidial wall pseudoparenchymatous, composed of oblong to isodiametric cells, 3–5 cell layers, 13–18 μm thick. Conidiogenous cells phialidic, smooth, hyaline, ampulliform to doliiform, 4.5–9.5 × 4–5 μm. Chlamydospores intercalary or terminal, pale brown, smooth, single or in chains, globose to oval. Conidia ellipsoidal to oblong, aseptate, hyaline, smooth, thin-walled, guttulate, 3–5.5 × 1.5–2.5 μm.

Culture characteristics: Colonies on PDA, 50–55 mm diam. after seven days of cultivation in the dark at 28°C, raised, margin regular, velvety, with abundant aerial mycelium, dense, white, pale yellow near the center; reverse: white to pale yellow, sienna pigment produced near the center.

Material examined: China, Guizhou Province, Guiyang City, from healthy stems of *R. roxburghii* (Rosaceae), 22 April 2020, H. Zhang (HGUP191049); living cultures were deposited in the Culture Collection at the Department of Plant Pathology, College of Agriculture, Guizhou University, China, No. GUCC 191049.1 and China General Microbiological Culture Collection Center, No. CGMCC 40110.

Notes: The screened strain HGUP191049 and the type of *Ep. latusicollum* are phylogenetically similar as they cluster together with well support (**Supplementary Figure 1**). Our collection resembles the type CGMCC 3.18346 in having a pycnidial wall, conidiogenous cells, and conidia. However, our collection slightly differs from the type in having aggregated conidiomata rather than solitary conidiomata (Chen et al., 2017). Therefore, the examined morphology overlaps and is phylogenetically identical to *Ep. latusicollum*. We report our collection as a new host record of *Ep. latusicollum* from the stem of *R. roxburghii*.

### 3.5 Genome sequencing and annotation

**Figure 5:**
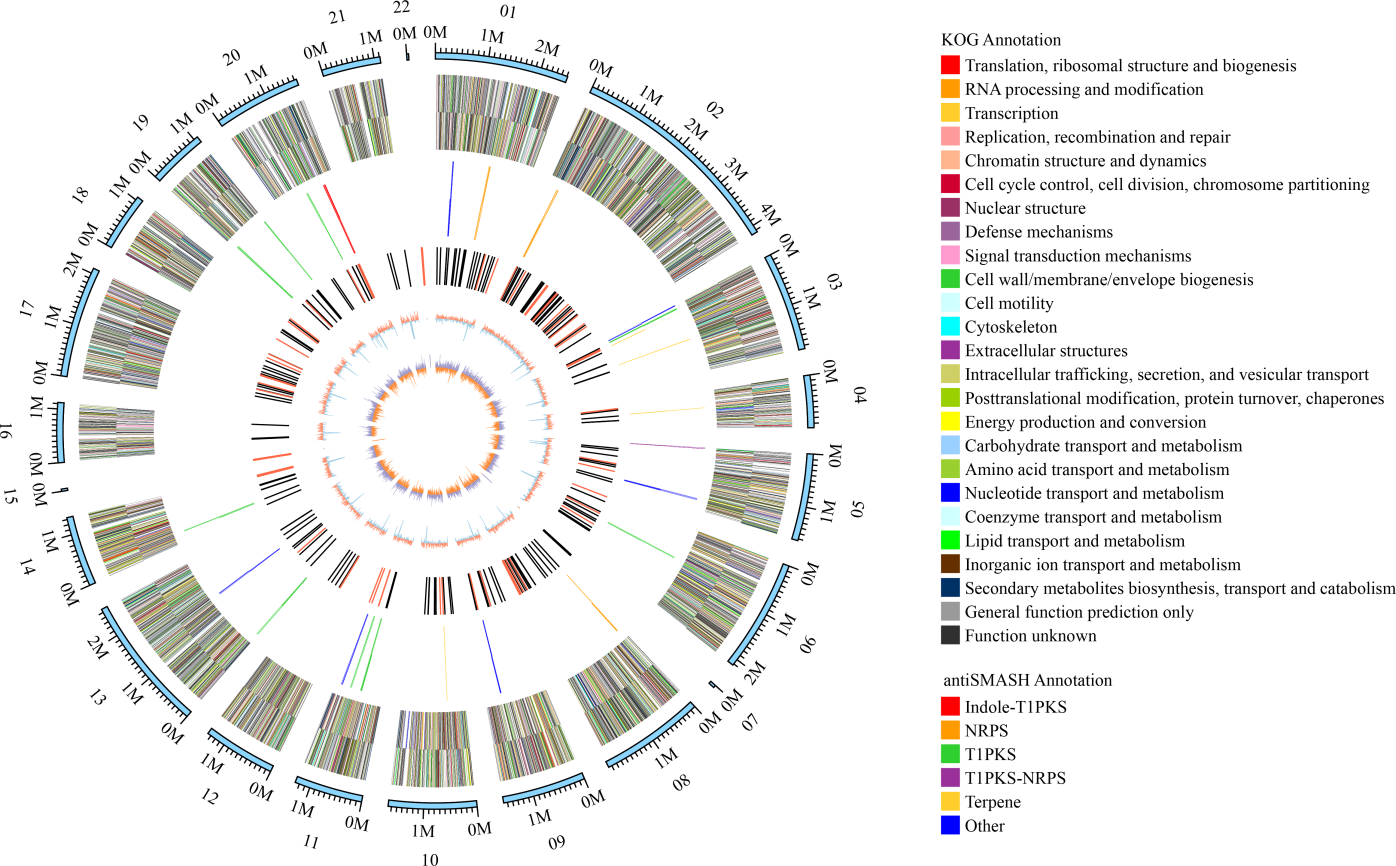
Circular map of genomic features of *Epicoccum latusicollum* HGUP191049. The peripheral circles represent the scaffolds (Mb scale), consisting of 22 scaffolds. From outer to inner circles (second to the sixth circle) are KOG annotation (forward and reverse strands), different colors indicate different functional classification; antiSMASH annotation (forward and reverse strands), different colors indicate different types of biosynthetic gene clusters (BGCs); ncRNA (black indicates tRNA, red indicates rRNA); GC content (red indicates greater than the mean, blue indicates less than the mean); GC skew (used to measure the relative content of G and C, GC skew = (G-C)/(G+C); purple indicates greater than 0, orange indicates less than 0).

Genome sequencing of *Ep. latusicollum* HGUP191049 was conducted using a combination of single molecule real-time (SMRT) and Illumina sequencing technologies. The obtained genome of HGUP191049 was assembled into 22 scaffolds, about 33.24 megabase pairs (Mbp), and 10,500 genes (**Figure 5**; **Table 2**). The estimated genome size of HGUP191049 is broadly congruent with other estimates of genome size in *Epicoccum*, 33–35 Mbp (**Supplementary Figure 2**) (Fokin et al., 2017; Oliveira et al., 2017; Guo et al., 2021). The N50 and N90 length of the scaffolds were 1,859,063 bp and 1,112,482 bp, respectively. The GC content was 52.06% for the genome and 54.82%% for the coding sequences. In total, 10,310 protein-encoding genes were predicted from the genome assembly. Among them, 10,197, 9,523, 5,981, and 4,530 genes have functional annotations in the Nr, KEGG, SwissProt, and KOG databases, respectively. In this study, 325 genes (3.10%) were associated with secondary metabolite biosynthesis, transport, or catabolism in the KOG database (**Supplementary Table 3**).

**Table 2:** Genome features of *Epicoccum latusicollum* HGUP191049.

Genome features	Value	Genome features	Value
Size of assembled genome (Mbp)	33.24	Protein-coding genes (≥ 60 aa)	10,304
GC content of assembled genome (%)	52.06	Min protein length (aa)	52
Number of scaffolds	22	Max protein length (aa)	9,186
N50 Length (bp)	1,859,063	tRNA genes	206
N90 Length (bp)	1,112,482	rRNA genes	88
Maximum length (bp)	4,138,377	Depth	295X
Minimum length (bp)	38,673	Genes assigned to KOG categories	4,530
Average gene length (bp)	1422.07	Total length of contigs	33242988
All protein-coding genes	10,310	Putative biosynthetic gene clusters for secondary metabolites	24

**Figure 6:**
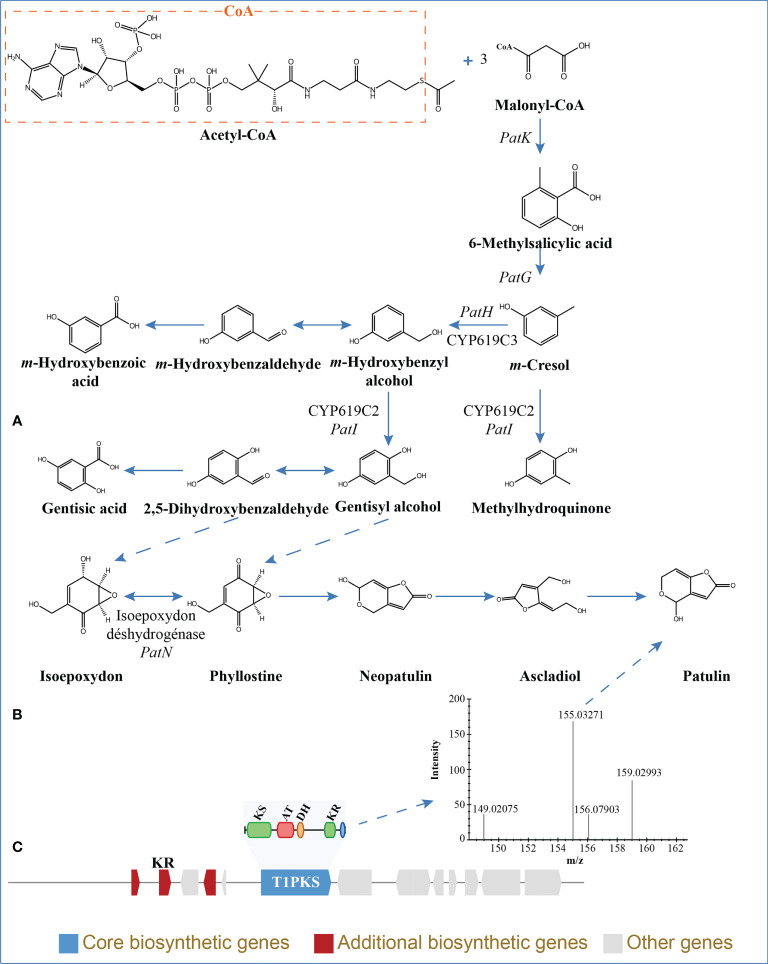
Patulin in *Epicoccum latusicollum* HGUP191049. **(A)** Biosynthetic pathways of patulin (Puel et al., 2010; Nielsen et al., 2017). **(B)** Non-targeted metabolic profiling spectrum. **(C)** Schematic representation of the putative BGC of patulin (cluster 16). KS, ketosynthase; AT, acyl transferase; DH, dehydratase; KR, ketoreductase; T1PKS, type I polyketide synthases.

The BGCs were analyzed using antiSMASH, and a total of 24 putative natural product BGCs of HGUP191049 were yielded, including three NRPSs, nine T1PKSs, four terpene synthases, one NRPS-T1PKS, one Indole-T1PKS, and six NRPS-like gene clusters (**Supplementary Table 4**). Of the 24 annotated BGCs, eight BGCs were found to share similarities in gene content with previously identified, while the remaining showed no significant similarities with currently known. These unknown BGCs could potentially shed light on the search for novel compounds. The antiSMASH and BLAST bioinformatics analyses identified three complete BGCs encoding dimethylcoprogen, (-)-mellein, and melanin. Other annotated potential products were squalestatin S1 (40% similarity), phomasetin (40%), oxyjavanicin (25%), patulin (20%), and azanigerone A (26%), respectively. Among these putative natural products, oxyjavanicin (**Supplementary Figure 3**), squalestatin S1 (**Supplementary Figure 4**), and patulin (**Figure 6**) have been reported to exhibit antimicrobial activity (Nicolaou et al., 1994; Paytubi et al., 2017; Kato et al., 2020). In this study, the putative patulin BGC is cluster 16 (T1PKS), sharing only 20% similarity to BGC0000120. We detected this compound in the secondary metabolites of *Ep. latusicollum* HGUP191049 (**Figure 6**).

### 3.6 Non-target metabolomics

**Figure 7:**
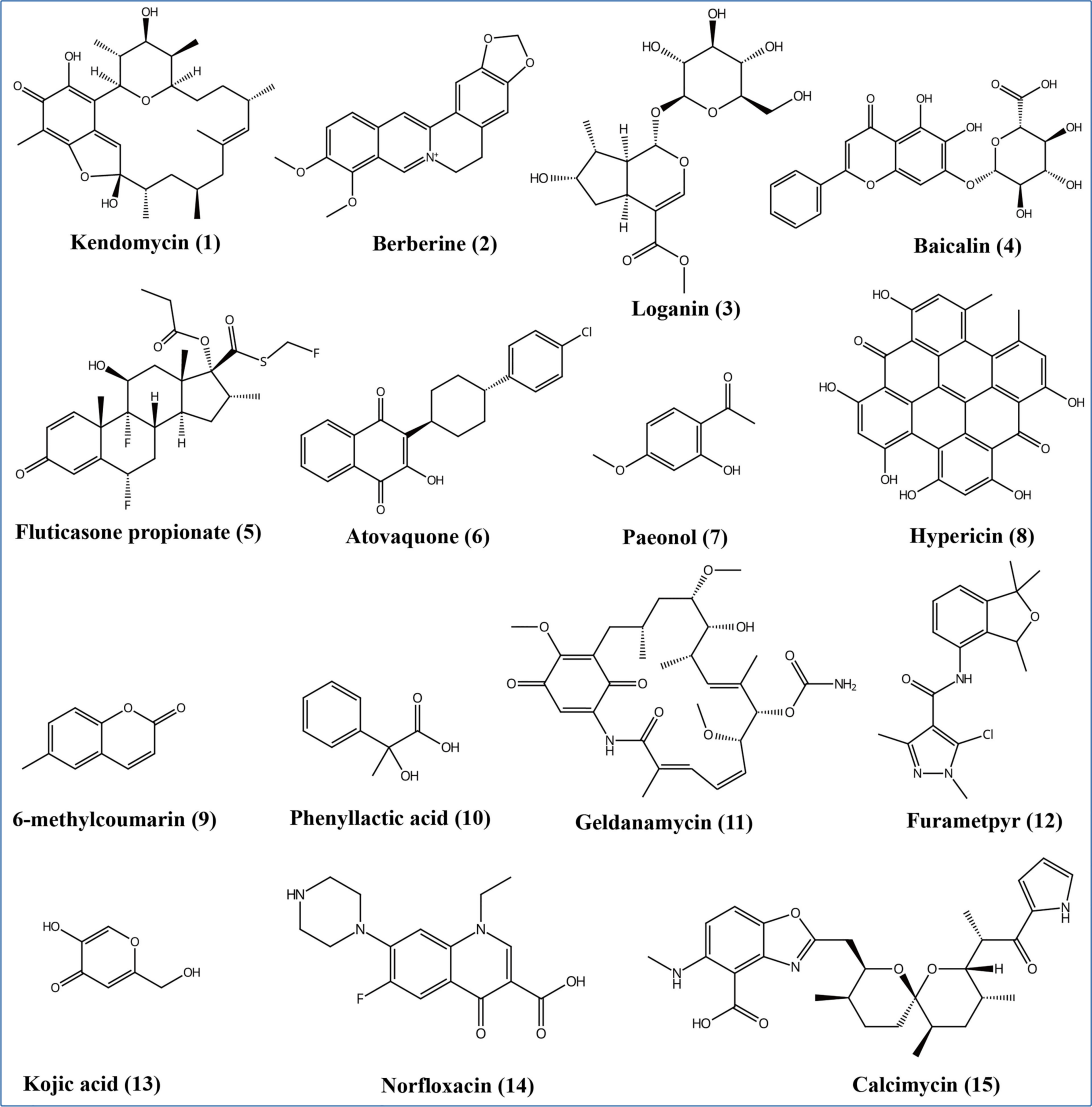
Some different structural types of antimicrobial secondary metabolites from *Epicoccum latusicollum* HGUP191049.

According to the non-target metabolomics profiling, we detected 999 metabolites for positive mode, 523 for negative mode, and 18286 (92.3%) unknown metabolites (positive and negative modes), which indicated that *Ep. Latusicollum* HGUP191049 might produce a large number of new compounds. It was determined by conducting a literature search whether known metabolites had antimicrobial activity. The results revealed about 120 compounds with antimicrobial activity, 7.9% of the known compounds (**Supplementary Table 5**). Specifically, some antimicrobial compounds with different structures were illustrated in **Figure 7**, including polyketide (e.g., kendomycin), alkaloids (e.g., berberine), terpenoids (e.g., geniposidic acid), flavonoids (e.g., nevadensin), steroids (e.g., fluticasone propionate), naphthoquinone compounds (e.g., atovaquone), anthraquinones (e.g., hypericin), phenolic compounds (e.g., mangostine), coumarin compounds (e.g., 6-methylcoumarin), fatty acid compounds (e.g., phenyllactic acid), carbamates (e.g., geldanamycin), amides (e.g., benzamide), heterocyclic compounds (e.g., kojic acid), antibiotic compounds (e.g., norfloxacin), and other antimicrobial compounds (e.g., (+)-trans-chrysanthemic acid, (S)-(-)-citronellic acid, and azadirachtin A). Within this, flavonoids are one of the most abundant groups of antimicrobial secondary metabolites. The highly structural diversity demonstrated that *Ep. latusicollum* HGUP191049 is a talented producer of antimicrobial compounds.

### 3.7 Comparative genomics analyses results

#### 3.7.1 Prediction and comparative analyses of pathogenicity-related genes

**Figure 8:**
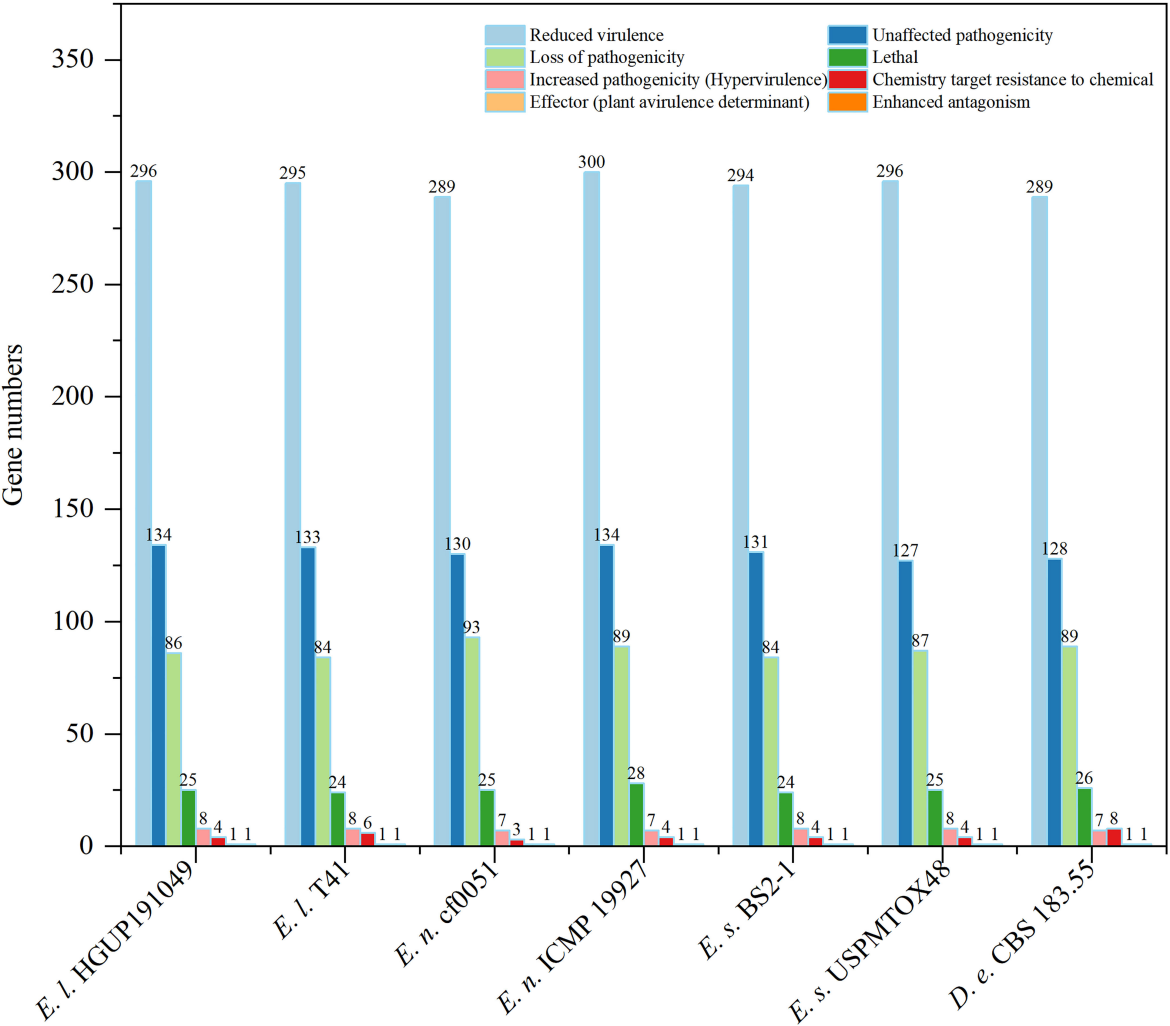
Comparisons and annotations of pathogen-host interactions (PHI). *D.e*, *Didymella exigua*; *E.l*, *Epicoccum latusicollum*; *E.n*, *Epicoccum nigrum*; *E.s*, *Epicoccum sorghinum*.

To identify and compare potential protein-coding genes related to pathogenicity and virulence in the genomes, whole genome blast analyses were performed against the pathogen-host interaction (PHI) gene database v. 4.13 at E<1*10^−20^ and identity≥70% (Prasad et al., 2015; Urban et al., 2017; Xu et al., 2020). Screening of PHI annotated phenotypes showed that most genes belonged to “reduced virulence”, “unaffected pathogenicity”, and “loss of pathogenicity”. In contrast, few genes were associated with the “effector (plant avirulence determinant)” (one gene), “enhanced antagonism” (one gene), and “chemistry target sensitivity to chemical” (none) phenotypes (**Figure 8**). Genes of the “increased pathogenicity (Hypervirulence)” type are key pathogenic ones. As illustrated in **Figure 8** and **Supplementary Figure 2**, strains with different nutrient modes of the same *Epicoccum* species may possess the same gene numbers of “increased pathogenicity (hypervirulence)” type, such as *Ep. latusicollum* (HGUP191049 and T41), *Ep. nigrum* (cf0051 and ICMP 19927), and *Ep. sorghinum* (BS2-1 and USPMTOX48), having 8, 7, 8 genes of this type for them, respectively. Of this phenotypic gene, the seven genomes in this study shared seven identical genes of this type, whereas *Ep. latusicollum* (HGUP191049 and T41) and *Ep. sorghinum* (BS2-1 and USPMTOX48) had one more of this type gene than the other genomes, namely PHI:5494, which may be a vital contributor to the difference in pathogenicity between species. In addition, an endophyte of the same species may contain more “loss of pathogenicity phenotypic genes” than a pathogen, e.g. endophytic strain HGUP191049 has two more genes of this type than pathogenic strain T41, and endophytic cf0051 has four more genes than pathogenic ICMP 19927. Moreover, for *Ep. latusicollum*, endophytic HGUP191049 had six “loss of pathogenicity” phenotypic genes (PHI: 2145, PHI: 4095, PHI: 10527, PHI: 9899, and PHI: 8875) different from the pathogenic T41 (PHI: 8734, PHI: 5232, and PHI: 9357), which may be one of the factors contributing to their differences in pathogenicity within species.

#### 3.7.2 Prediction and comparative analyses of carbohydrate-active enzyme genes

Carbohydrate-active enzymes (CAZymes) are essential for fungal biological activity. CAZymes are responsible for degrading host plant cells and establishing colonization for plant pathogenic and endophytic fungi. As biocontrol fungi, CAZymes can be used to destroy the cell walls of pathogens and nematodes (Yang et al., 2019). The CAZymes involved in the degradation of plant cell walls were further classified into the degradation of cellulose, hemicellulose, and pectin, and those involved in the degradation of fungal cell walls were grouped into the degradation of chitin and β-1,3-glucan (Zhao et al., 2013; Kubicek et al., 2014; Yang et al., 2019).

**Figure 9:**
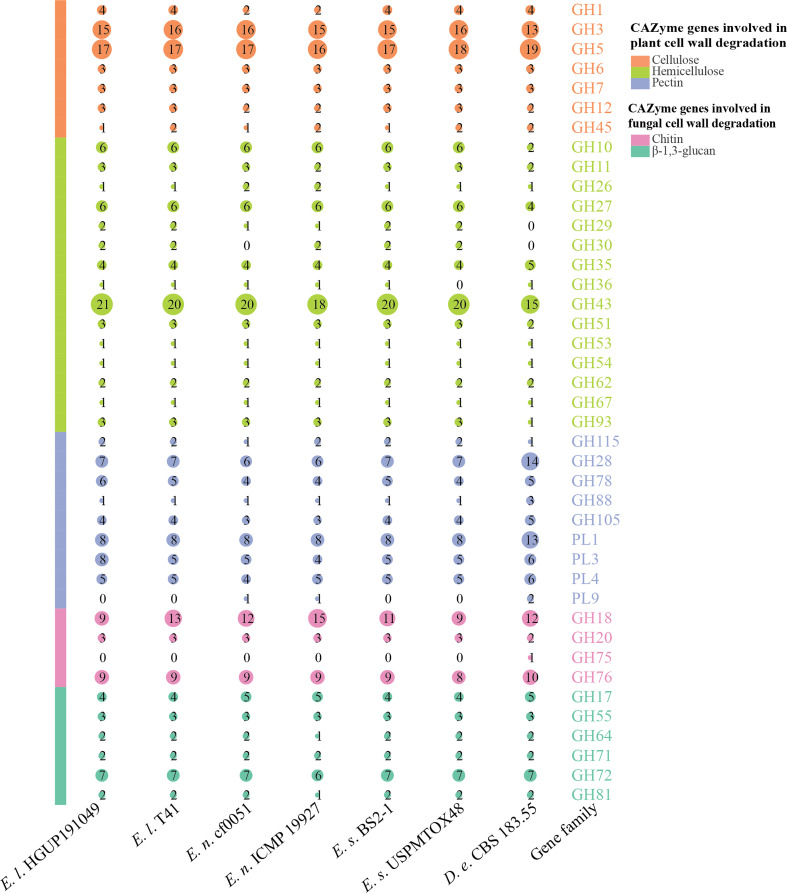
Comparisons and annotations of carbohydrate-active enzyme genes. *D.e*, *Didymella exigua*; *E.l*, *Epicoccum latusicollum*; *E.n*, *Epicoccum nigrum*; *E.s*, *Epicoccum sorghinum.* Different circle sizes indicate the number of different gene families.

As can be derived from **Figure 9**, the main CAZyme gene families that differ significantly between *Epicoccum* and *Didymella* are GH10, GH28, GH43, and PL1. In this study, 41.5% (17/41) of the families are identical among and within species in *Epicoccum*, such as GH6, all of which are 3 in number. Other families differ in the number of characteristics by 1–2, with a few 3, as in GH43. However, it is significantly different for GH18, which belongs to a family associated with chitin degradation, with numbers ranging from 9 to 15, which may be an important factor influencing the difference in the antifungal potential of *Epicoccum* spp. Of *Ep. latusicollum*, the biological activity of strain HGUP191049 distinguished from T41 in having different amounts of GH3 and GH45, GH43, GH78 and PL3, and GH18, for the degradation of cellulose, hemicellulose, pectin, and chitin, respectively.

#### 3.7.3 Prediction and comparative analyses of BGCs

**Figure 10:**
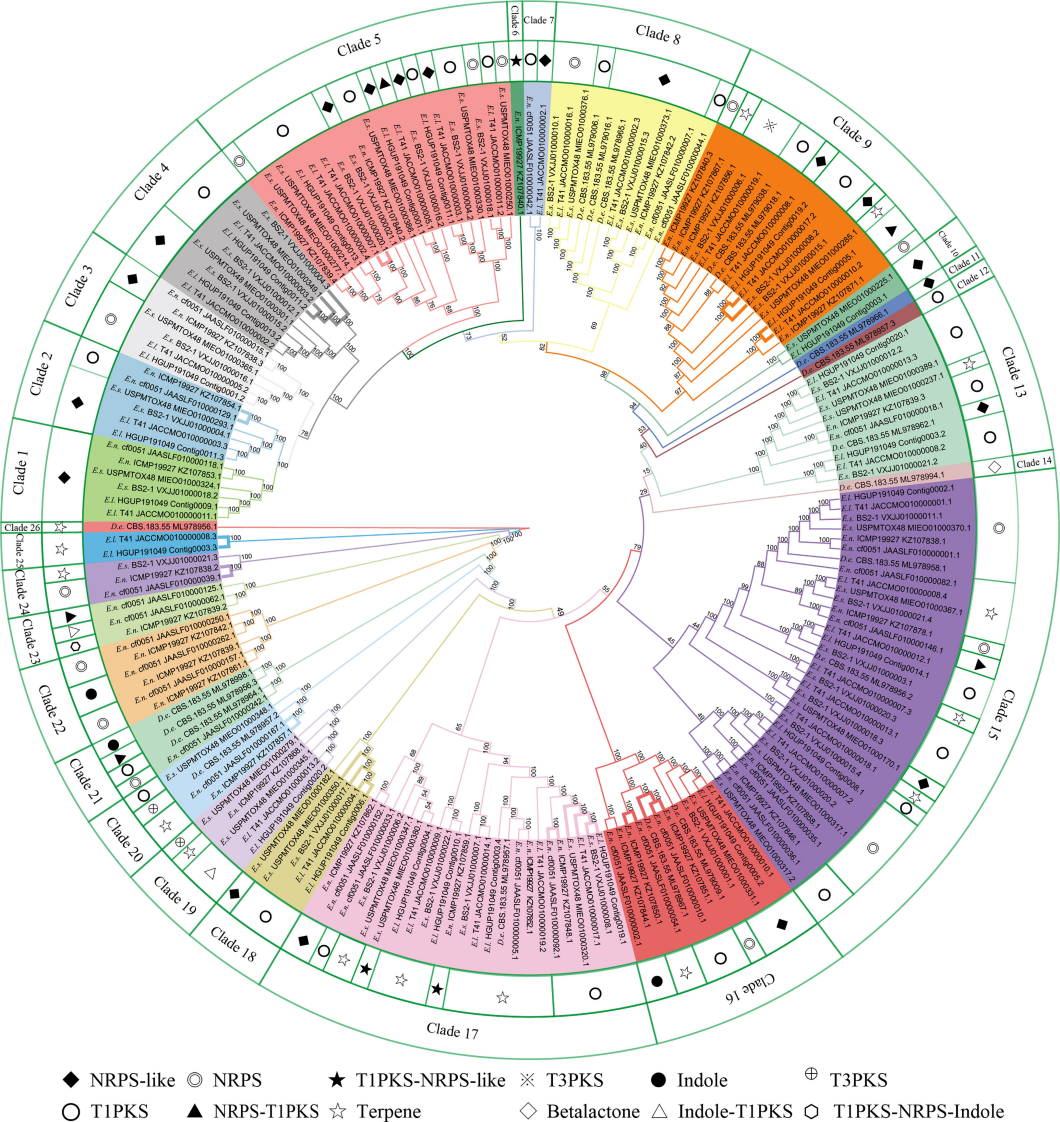
Phylogenetic analyses of biosynthetic gene clusters (BGCs). Bootstrap support values for maximum likelihood are given near nodes. *D.e*, *Didymella exigua*; *E.l*, *Epicoccum latusicollum*; *E.n*, *Epicoccum nigrum*; *E.s*, *Epicoccum sorghinum.* The species name is followed by the strain number, and the final number indicates the gene of each BGC. **Bolded** adjacent branches indicate coding for the same compound.

In this study, there were 177 BGCs from six *Epicoccum* genomes, of which PKS accounted for 35.0%, NRPS for 18.6%, terpene for 15.8%, hybrid PKS/NRPS for 6.8%, indole for 2.3%, and other unknown BGCs (NRPS-like) for 21.5% (**Figure 10**), which suggests that *Epicoccum* is a promising source of terpenes besides the traditional PKS- and NRPS-encoded compounds.

Phylogenetic relationships of BGCs from six *Epicoccum* strains and evolutionarily adjacent species *D. exigua* were analyzed to investigate differences among secondary metabolites of *Epicoccum* spp. (**Figure 10**). The result showed that BGCs could be grouped into 26 clades. The same types of BGCs with high identity may encode the same secondary metabolites, while the corresponding BGCs of a compound may be in different evolutionary branches. Notably, *Epicoccum* species have BGCs encoding the same compounds. The same branch of *Ep. latusicollum* (Contig0006.1 and JACCMO010000004.1) and *Ep. sorghinum* (VXJJ01000017.1 and MIEO01000350.1) (Clade 18), which all encode oxyjavanicin, where *Ep. nigrum*, the BGC JAASLF010000044.1, which encodes this compound, belongs to Clade 8. Similarly, squalestatin S1 is also encoded by BGCs from six different *Epicoccum* genomes. Consequently, it is presumed that oxyjavanicin and squalestatin S1, both of which have been reported as antimicrobial agents, are secondary metabolites shared by *Epicoccum* spp.

## 4 Discussion

Medicinal plants have long been used as a source of medicine. Approximately 8,000 medicinal plants have been developed into drugs and biocides, contributing more than 7,000 compounds to the pharmaceutical industry (Kaul et al., 2012). However, the overuse of medicinal plants in traditional folk medicine practices has led to environmental degradation and loss of biodiversity. Developing bioactive compounds based on endophytic fungi can reduce deforestation and the extinction of important and valuable medicinal plants (Uzma et al., 2019). Medicinal plants are a valuable source for exploring biologically active endophytes (Kaul et al., 2012). In this study, *R. roxburghii* is an economically important source of medicine and food. Its fruit is rich in vitamin C (up to 2 000 mg/100 g), superoxide dismutase (SOD), and flavonoids (Xu et al., 2019; Hou et al., 2020). The root, leaf, and fruit of *R. roxburghii* have been used as traditional medicinal materials to treat several diseases, such as dyspepsia, enteritis, and scurvy. In addition, some components extracted from *R. roxburghii* have been demonstrated to possess biological activities, including hypoglycemic, hypolipidemic, immune-enhancing, and antitumor effects (Zhang et al., 2021b). More importantly, *R. roxburghii* is also a plant source of antimicrobial compounds (Ma et al., 2020; Wang et al., 2021). Therefore, we selected *R. roxburghii* as a candidate for screening endophytic fungi with antimicrobial activity.

Species-level identification of fungi is a critical step to ensure reproducibility and is essential for both basic scientific research (ecology, taxonomy) and applied scientific research (genomics, bioprospecting). However, only 14% of fungal secondary metabolites studies have combined morphological and molecular data for identification (Raja et al., 2017). The results of these investigations suggest that the identification of fungi in most such studies is unreliable, as a single gene (mainly ITS) may fail to distinguish closely related members of certain genera phylogenetically. More than a quarter of GenBank fungal ITS sequences have not been adequately confirmed taxonomically (Zhang et al., 2021b). For accurate species identification, molecular data (preferably polygenic) should be combined with morphological studies (Woudenberg et al., 2017). In this study, we obtained the antimicrobial active strain *Ep. latusicollum* HGUP191049, whose taxonomic status was confirmed by morphology and multigene phylogenetic analyses.

In this study, the strains isolated from *R. roxburghii* with antimicrobial activity were screened out by multigene phylogenetic analyses (ITS, LSU, RPB2, TUB, TEF, and ACT), the plate confrontation method, and the disc diffusion method, namely *Ep. latusicollum* HGUP191049, *Neofusicoccum* sp. HGUP191080, and *Se. terrestris* HGUP190028. *Epicoccum latusicollum* has been reported to be capable of causing several plant diseases, including leaf spots on tobacco and *Elaeagnus pungens* (Guo et al., 2021; Qi et al., 2021), stalk rot on maize (*Zea mays* L.) (Xu et al., 2022), and root rot on *Nicotiana tabacum* (Gai et al., 2020). In this work, this species is first reported as an endophytic fungus with antimicrobial activity and is a new host record from *R. roxburghii*. Another strain with antimicrobial activity, *Neofusicoccum* sp. HGUP191080 may phylogenetically represent a new species and requires further identification by morphology. *Neofusicoccum* species, which are endophytes or pathogens of plants, produce structurally different metabolites that show interesting biological activities such as antibacterial, cytotoxic, and phytotoxic (Salvatore et al., 2021). Finally, *Se. terrestris* caused pink root rot in various plants, such as squash, canola, and winter squash (Ikeda et al., 2012; Yang et al., 2017; Rivedal et al., 2018). However, as an endophyte isolated from *Dysoxylum binectariferum*, *Se. terrestris* is known to produce blennolides with anticancer and antimicrobial activity (Arora et al., 2018). Thus, some species commonly reported as pathogens may have potential biological activity as endophytic fungi. Further MIC assays demonstrated that the present study’s antimicrobial strength and spectrum of *Ep. latusicollum* HGUP191049 were superior to other strains.

The development of genomics, transcriptomics, proteomics, metabolomics, high-throughput technologies, and computational resources has significantly broadened the understanding of the key pathways affecting the synthesis of fungal secondary metabolites (Palazzotto and Weber, 2018). In this study, genomics, non-target metabolomics, and comparative genomics were performed further to investigate the biosynthetic capacity of *Ep. latusicollum* HGUP191049. Genes required for secondary metabolite synthesis are typically arranged in a multigene biosynthetic gene cluster (Yang et al., 2019). With this high-quality genome sequence and annotation, we predicted a total of 24 BGCs, which may encode eight known compounds. Of these compounds, squalestatin S1, oxyjavanicin, and patulin were reported to have antimicrobial activity (Nicolaou et al., 1994; Paytubi et al., 2017; Kato et al., 2020). Genetic modification of BGCs and/or introduction of a particular mutation provides opportunities to obtain derivatives of the original metabolites (Ichikawa et al., 2012). Genome mining of gene clusters encoding biosynthetic pathways of fungal secondary metabolites has become a critical approach for new compound discovery (Weber et al., 2015). The sequencing and annotation of the *Ep. latusicollum* HGUP191049 genome is the foundation for the identification of antimicrobial compound BGCs, the activation of silencing gene clusters, and the identification and regulation of biosynthetic pathways. In this study, non-targeted metabolic analyses further revealed the biosynthetic capacity and potential antimicrobial compounds of *Ep. latusicollum* HGUP191049 by determining all detectable metabolites. Moreover, patulin, a compound encoded by gene cluster 16, has also been detected.

Comparative genomics aims to use an ensemble of related genomes to improve the understanding of each genome in the set (Haubold & Wiehe, 2004). *Epicoccum* is a genus in which endophytic, saprophytic, and pathogenic modes of nutrition coexist, such as *Ep. nigrum* is a primary saprophyte involved in the retting of flax (Brown, 1984), an endophytic fungus isolated from the leaves of *Lysidice rhodostegia* (Wang et al., 2010), even a pathogen that causes leaf spot disease on *Lablab purpureus* (Mahadevakumar et al., 2014). So, *Epicoccum* species may reshape their lifestyles among endophytic, saprophytic, and pathogenic to adapt to changing environmental conditions (Kuo et al., 2014). The fungus may secrete numerous proteins that facilitate colonization during interaction with the plant (Yin et al., 2015). Nine high-level phenotypic terms are defined in PHI-base to compare the pathogen-host interactions between organisms across the tree of life (Urban et al., 2017). Using comparative genomic approaches, we show that PHI:5494, one of the “increased pathogenicity (hypervirulence)” type genes, may be an important factor in the difference in pathogenicity between *Epicoccum* species. Moreover, endophytic HGUP191049 had six “loss of pathogenicity” phenotypic genes different from the pathogenic T41, which may account for the lifestyle differences in *Ep. latusicollum*.

Currently, CAZyme gene families are defined and classified into six main categories in the CAZy database: glycosyltransferases (GTs), glycoside hydrolases (GHs), polysaccharide lyases (PLs), carbohydrate esterases (CEs), carbohydrate-binding modules (CBMs), and enzymes of auxiliary activities (AAs) (Zhang et al., 2018). Of these families, GH18 is related to a family of chitin degradation in amounts ranging from 9 to 15 by comparative analyses, which may be an important factor contributing to the differences in the antifungal potential of *Epicoccum* spp.

The secondary metabolites of fungi constitute a rich source of natural products with antimicrobial activity. Genes encoding biosynthetic pathways of secondary metabolites are usually located on chromosomes forming BGCs (Yao et al., 2021). Results from comparative analyses show that *Epicoccum* is a promising source of terpenes. Terpenes exhibit antimicrobial activity owing to their highly lipophilic nature, which may interfere with the integrity and function of cell membranes (Sohrabi et al., 2015). In addition, oxyjavanicin and squalestatin S1 reported as antimicrobial agents (Nicolaou et al., 1994; Kato et al., 2020), are putative secondary metabolites shared by *Epicoccum* spp. In this study, a broad-spectrum antimicrobial potential strain was screened out from the endophytic fungi of *R. roxburghii* and analyzed for biosynthetic capacity.

## 5 Conclusion

We isolated 54 endophytic fungi from *R. roxburghii* and analyzed their multigene phylogenetic relationships. *In vitro* antimicrobial experiments revealed that the endophytic strain with broad-spectrum antimicrobial potential, *Ep. latusicollum* HGUP191049, was screened out. Multi-omics analyses suggested that *Epicoccum* spp. is an ideal source of antimicrobial compounds. In conclusion, plants with specific medicinal value are promising sources for isolating endophytes with corresponding particular functions.

## Data availability statement

The datasets presented in this study can be found in online repositories. The accession numbers of the sequences deposited in GenBank are: ITS: MZ541933–MZ541986; LSU: MZ540051–MZ540080; RPB2: MZ546146–MZ546149 and OP321271-OP321292; TUB: MZ546150–MZ546167 and OP312077–OP312084; TEF1: MZ546168–MZ546171; ACT: MZ546172. The Ep. latusicollum HGUP191049 whole genome sequence data have been submitted to the GenBank database under accession no. JANURY000000000.

## Author contributions

All authors listed have made a substantial, direct and intellectual contribution to the work, and approved it for publication.

## Funding

This work is supported by the following projects: the Guizhou Provincial Science and Technology Projects (No. [2021]221 and [2020]1Y043), the Guizhou Province Science and Technology Innovation Talent Team Project (Qian Ke He Pingtai Rencai–CXTD [2021]004), and the Natural Science Foundation of China (No. 32060009).

## Acknowledgments

The authors thank Prof. Yan-Feng Han and Dr. Li Luo for their crucial help in sampling. We also thank Prof. Zhong Li, Associate Prof. Xin Xie, Associate Prof. Zhi-Bo Zhao, Associate Prof. Hai-Xia Ding, Dr. Xian-Feng Hu, and Ying Shen for providing the tested strains.

## Conflict of interest

The authors declare that the research was conducted in the absence of any commercial or financial relationships that could be construed as a potential conflict of interest.

## Publisher’s note

All claims expressed in this article are solely those of the authors and do not necessarily represent those of their affiliated organizations, or those of the publisher, the editors and the reviewers. Any product that may be evaluated in this article, or claim that may be made by its manufacturer, is not guaranteed or endorsed by the publisher.

## Supplementary material

Supplementary Figure 1 Phylogenetic analysis of *Epicoccum latusicollum* HGUP191049, phylogenetic tree generated from a maximum likelihood analysis based on the combined LSU, ITS, RPB2, and TUB sequence data, the tree is rooted with *Didymella exigua* CBS 183.55 and *D. rumicicola* CBS 683.79, **T** type or ex-type.

Supplementary Figure 2 Maximum likelihood phylogram and genome statistics of *Epicoccum* species analysed in this study. The genome accession numbers of *Ep. latusicollum* (HGUP191049 and T41), *Ep. nigrum* (cf0051 and ICMP 19927), *Ep. sorghinum* (BS2-1 and USPMTOX48), and *Didymella exigua* (CBS 183.55) are JANURY000000000, JACCMO000000000, JAASLF000000000, NCTX00000000, VXJJ00000000, MIEO00000000, and VOSY00000000, respectively.

Supplementary Figure 3 Putative oxyjavanicin in *Epicoccum latusicollum* HGUP191049. a. Oxyjavanicin structure b. Schematic representation of the putative BGC of oxyjavanicin (cluster 11). **TD**: thioesterase domain, **KS**: ketosynthase, **AT**: acyl transferase, **PT**: product template, **T1PKS**: type I polyketide synthases.

Supplementary Figure 4 Putative squalestatin S1 in *Epicoccum latusicollum* HGUP191049. **(A)** Squalestatin S1 structure **(B)** Schematic representation of the putative BGC of squalestatin S1 (cluster 4).
