# Supplementary figures and images for: Screening for broad-spectrum antimicrobial endophytes from Rosa roxburghii and multi-omic analyses of biosynthetic capacity
Source: Front Plant Sci. 2022 Nov 16;13:1060478. doi: 10.3389/fpls.2022.1060478 (PMC9709285; doi:10.3389/fpls.2022.1060478)

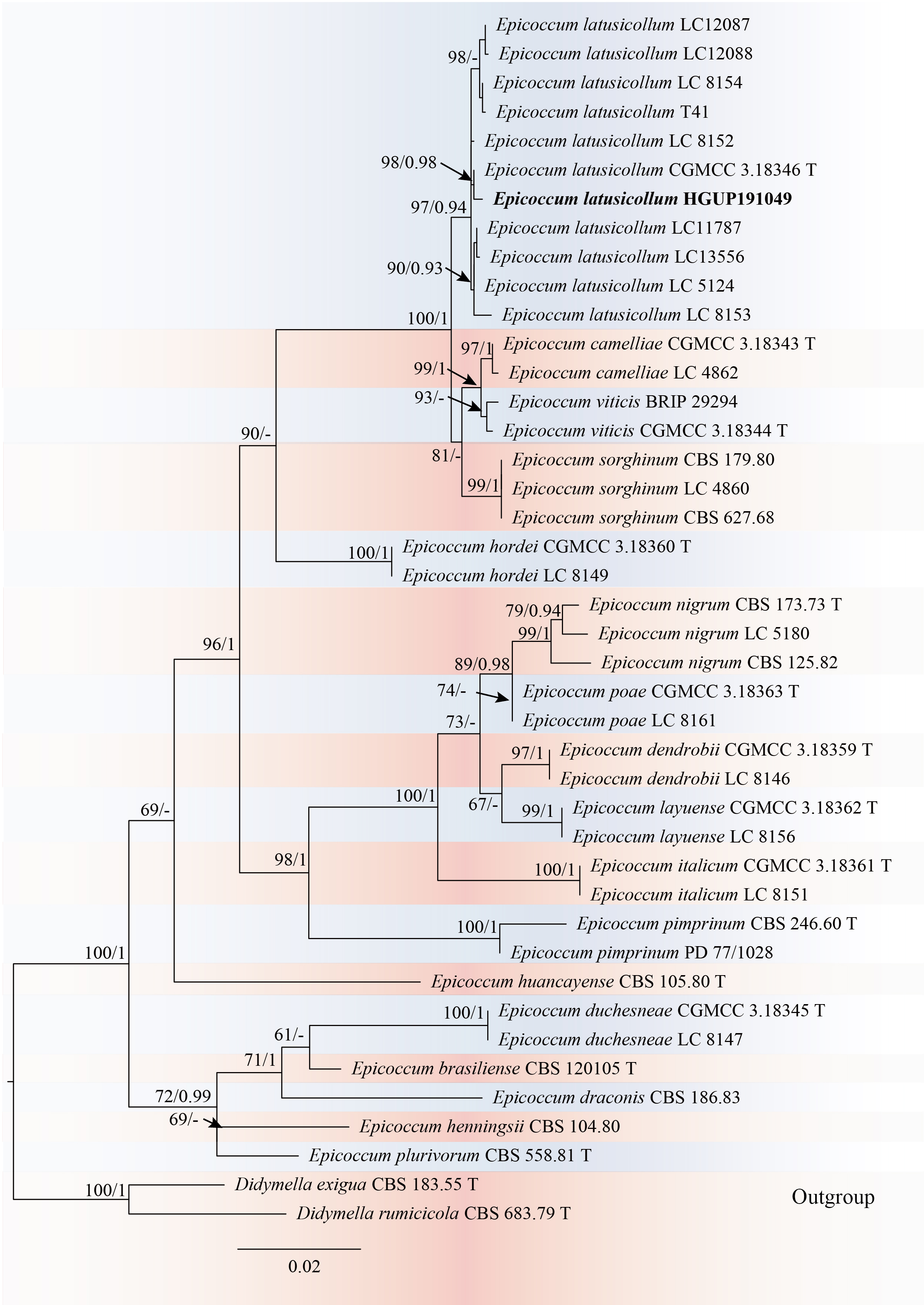

Supplement: Supplementary Figure 1 — Phylogenetic analysis of Epicoccum latusicollum HGUP191049, phylogenetic tree generated from a maximum likelihood analysis based on the combined LSU, ITS, RPB2, and TUB sequence data, the tree is rooted with Didymella exigua CBS 183.55 and D. rumicicola CBS 683.79, T type or ex-type. [file Image_1.jpeg]

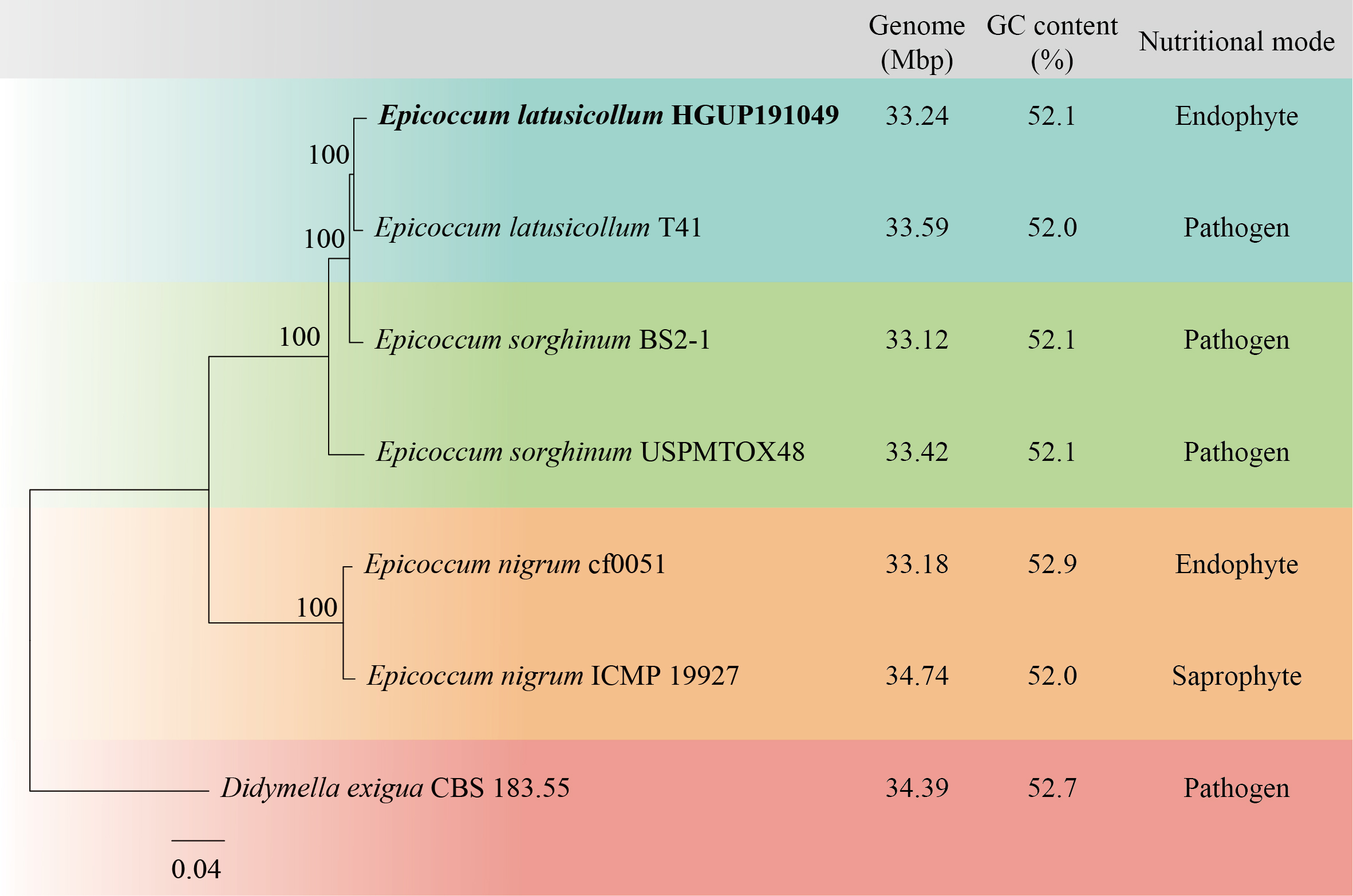

Supplement: Supplementary Figure 2 — Maximum likelihood phylogram and genome statistics of Epicoccum species analysed in this study. The genome accession numbers of Ep. latusicollum (HGUP191049 and T41), Ep. nigrum (cf0051 and ICMP 19927), Ep. sorghinum (BS2-1 and USPMTOX48), and Didymella exigua (CBS 183.55) are JANURY000000000, JACCMO000000000, JAASLF000000000, NCTX00000000, VXJJ00000000, MIEO00000000, and VOSY00000000, respectively. [file Image_2.jpeg]

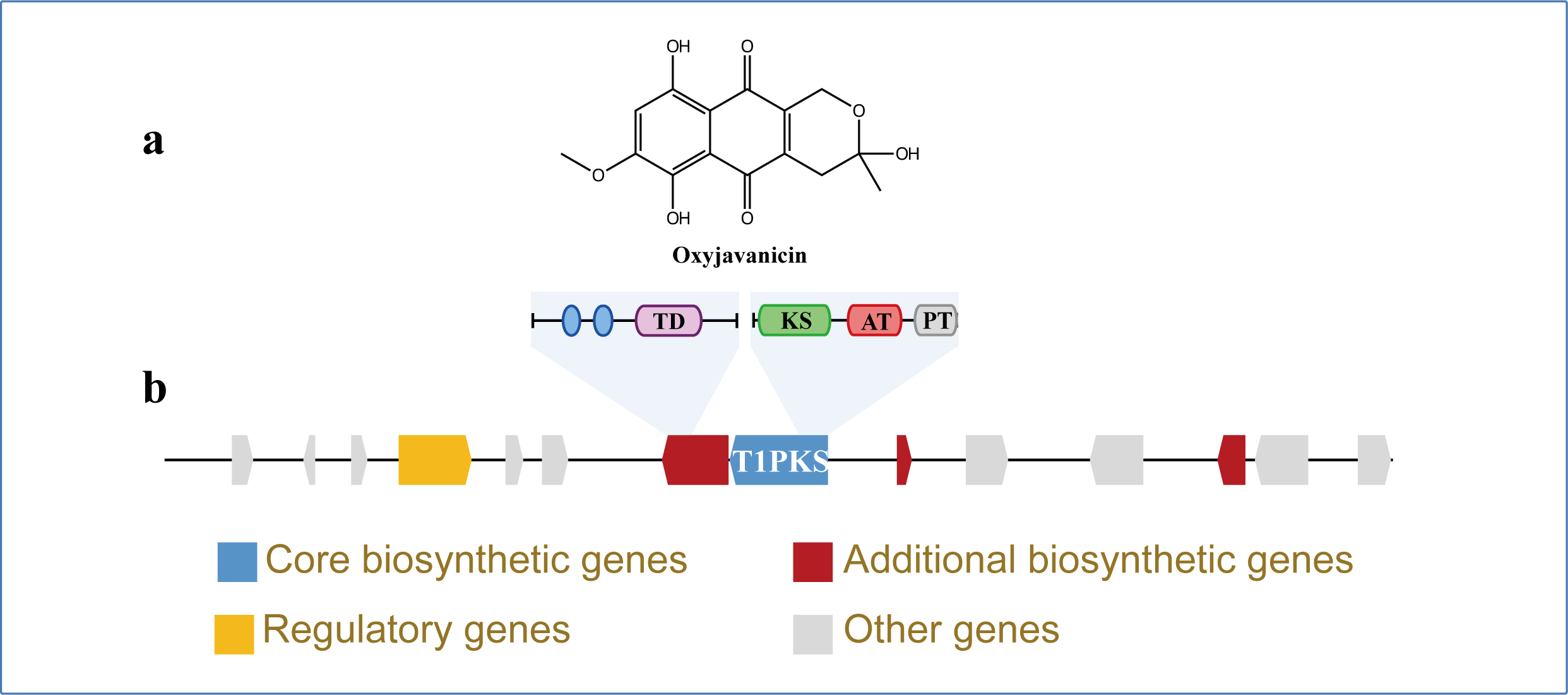

Supplement: Supplementary Figure 3 — Putative oxyjavanicin in Epicoccum latusicollum HGUP191049. a. Oxyjavanicin structure b. Schematic representation of the putative BGC of oxyjavanicin (cluster 11). TD: thioesterase domain, KS: ketosynthase, AT: acyl transferase, PT: product template, T1PKS: type I polyketide synthases. [file Image_3.tif]

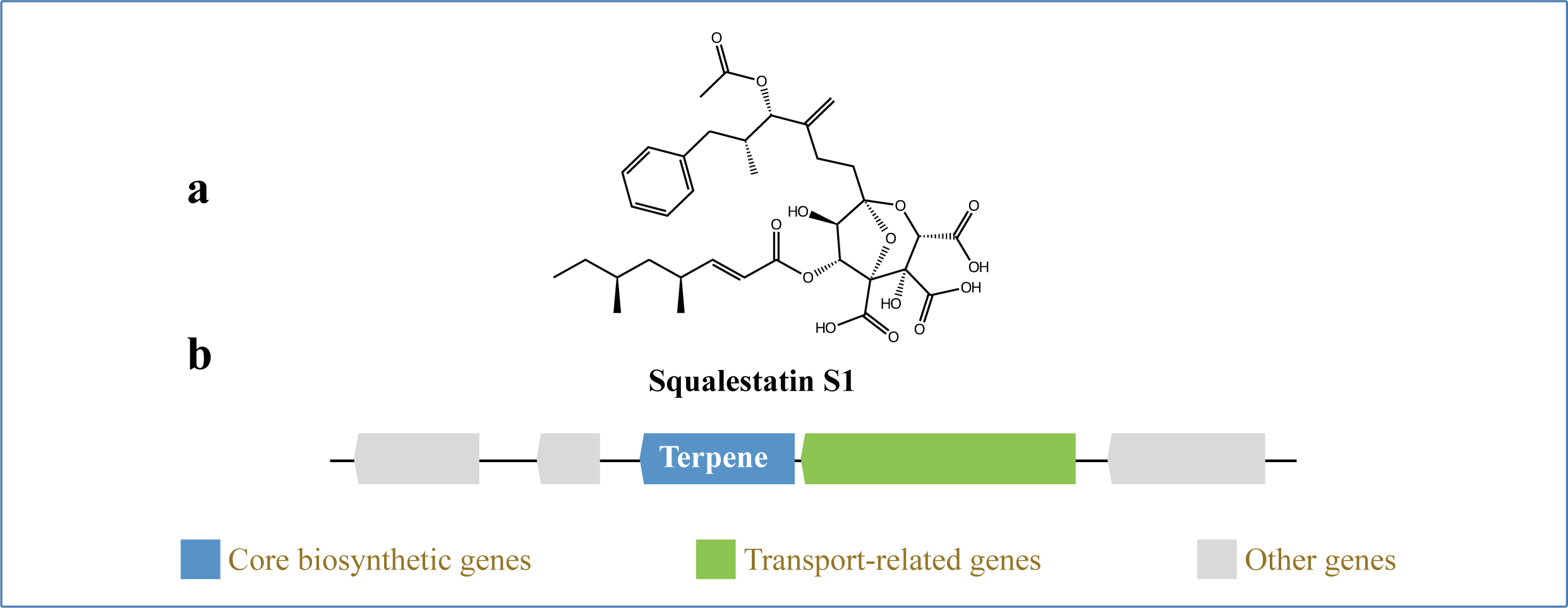

Supplement: Supplementary Figure 4 — Putative squalestatin S1 in Epicoccum latusicollum HGUP191049. (A) Squalestatin S1 structure (B) Schematic representation of the putative BGC of squalestatin S1 (cluster 4). [file Image_4.tif]
